# Editorial: Mapping Human Sensory-Motor Skills for Manipulation Onto the Design and Control of Robots

**DOI:** 10.3389/fnbot.2019.00001

**Published:** 2019-01-22

**Authors:** Matteo Bianchi, Gionata Salvietti

**Affiliations:** ^1^Research Center “Enrico Piaggio”, University of Pisa, Pisa, Italy; ^2^Department of Information Engineering, University of Pisa, Pisa, Italy; ^3^Department of Information Engineering and Mathematics, University of Siena, Siena, Italy; ^4^Department of Advanced Robotics, Istituto Italiano di Tecnologia, Genoa, Italy

**Keywords:** human hands, robotic hands, human-robot interfaces, human arm, human touch, prosthetics

The extraordinary human sensory-motor capabilities arise from the interaction with the external world and the interplay of different elements, which are controlled within a space whose dimensionality is lower than the available number of dimensions, as suggested by the concept of synergies, see (e.g., Turvey, 2007; Latash, 2008; Santello et al., 2013). This general simplification approach has then been successfully used in robotics, to inform the development of simple yet effective artificial devices, see (e.g., Santello et al., 2016). Mutual inspiration between robotics and neuroscience could hence be the key to advance both these disciplines: through a bio-aware approach for the design of mechatronic systems, on one side, and the deployment of technical tools for novel neuroscientific experiments, on the other. The manuscripts presented in this e-book shed light on the organization of human sensory-motor architecture, presenting instruments and mechatronic systems that can be successfully applied to neuroscientific investigation. At the same time, we report on robotic translations of neuroscientific outcomes.

## Investigation of Human Sensory-Motor Behavior

In Averta et al. functional principal component analysis (fPCA) was applied, for a first time, to upper limb human actions, to unveil principal motor control schemes of arm joints. Results show that a combination of few principal time-dependent functions can explain most of trajectory variability in daily living activities. These findings can be applied for planning robotic manipulators and characterizing human upper limb kinematics in physiological and pathological conditions. The latter affects not only the motor components but also subjects' somato-sensation, whose assessment has received limited attention compared to motoric abilities. In Ballardini et al. a low-cost, bimanual mechatronic system is presented, which acts as a tactile stimulator and recorder. Results from tests with healthy subjects and post-stroke individuals show that the system can be a viable solution for characterizing tactile perceptual abilities at different body locations. The correct quantification of the performance of human somatosensory system can also provide useful inspiration for a successful human-robot interaction through haptic feedback. However, there are cases where the hands, which can be regarded as the organ of touch (Bicchi et al., 2011), are not accessible and other alternatives for haptic feedback delivery have to be investigated. Relying on the findings that humans can integrate normal force feedback at the toe into the sensorimotor loop, in Hagengruber et al. authors analyze human discrimination capabilities of spatial forces with different amplitudes and directions of application, at the bare front side of the toe. This provides a perceptual workspace that can be employed to design robotic devices for sensory substitution. Human afferences are not limited to touch, but they encompass multiple sensing channels, such as vision. Classic psychophysics characterizes sensory performance in terms of Weber's law and Just Noticeable Difference. However, the assumptions underneath these approaches can be violated in natural action-perception tasks, as it is the case of vision-guided grasping. Since perception and action are not synchronized in tele-robotic grasping, telerobotic systems can be an ideal platform to study the underlying causes that determine a violation of Weber's law. Afgin et al. propose a telerobotic system with time delays to investigate the perceptual basis of grasp control. White et al. study the modulation of the grip force during the interaction with soft and rigid virtual objects, when the stiffness is varied continuously across trials. Results suggest a sudden transition phase between two feedforward controllers, which is triggered at a given stiffness level.

## Bio-Aware Robotics And Man-Machine Interfaces

In Salvietti, the principal solutions for the design of robotic hands that implement the inter-joint coupling associated to the concept of hand synergies are reviewed. Synergistic inspiration has been also combined with soft robotics for a novel generation of deformable, robust, and functional artificial hands (Catalano et al., 2014). These end-effectors have attracted the attention of prosthesis designers, since they guarantee a simplified control and a natural interaction with the environment. Under this regard, promising results have been obtained with the SoftHand—Pro, SHP (Godfrey et al., 2017), an anthropomorphic, adaptable myo-prosthetic robotic hand with 19 DoFs but actuated using only one motor [controlled with two surface electromyographic (sEMG) electrodes]. To improve the SHP capabilities for fine grasp force control (Fu and Santello), propose a hybrid-gain myoelectric controller that switches the control gain based on the hand sensorimotor state. Haptic feedback was also delivered at the upper arm (Casini et al., 2015). Results show that the hybrid control architecture improves task completion speed and fine control, leading to performance qualitatively similar to the one of native human hands. The intrinsic capability of humans to vary the stiffness of their muscular-skeletal system is another key feature that allows complex motor behavior (Della Santina et al., 2017). Recently, mechanical structures with variable intrinsic stiffness have been proposed (Vanderborght et al., 2013) for energy-efficient action completion, as it could be the case of prostheses for cyclical drumming tasks. To achieve this goal, in Stillfried et al. able bodied drummers were asked to play simple regular drum beats. Results show that a series-elastic connection element between the forearm and the drumstick appears to lower the muscular effort of drumming, while a stiff connection seems to minimize the mental load and has a positive effect on the performance of drumming novices. In Zeng et al. an augmented reality AR guiding assistance method is presented, which enhances visual feedback to the user for a combined electroencephalography—based Brain Machine Interface (BMI) and eye tracking control of a robotic arm. Experimental results show that such a hybrid Gaze-BMI controller with the inclusion of AR information increases performance efficiency and reduces the cognitive load. In Fathaliyan et al. human gaze behavior and gaze–object interactions in 3D during a complex bimanual task are investigated. The goal is to extract salient features that can be fed to machine learning algorithms for human action recognition, with promising applications to assistive robots and robotic co-workers.

## Author Contributions

Both authors acted as guest editors for the related research topic. In the editorial, they have made a substantial, direct and intellectual contribution to the work, and approved it for publication.

### Conflict of Interest Statement

The authors declare that the research was conducted in the absence of any commercial or financial relationships that could be construed as a potential conflict of interest.
